# Mitochondrial Redox Metabolism in Trypanosomatids Is Independent of Tryparedoxin Activity

**DOI:** 10.1371/journal.pone.0012607

**Published:** 2010-09-08

**Authors:** Helena Castro, Susana Romao, Sandra Carvalho, Filipa Teixeira, Carla Sousa, Ana M. Tomás

**Affiliations:** 1 IBMC - Instituto de Biologia Molecular e Celular, Universidade do Porto, Porto, Portugal; 2 ICBAS - Instituto de Ciências Biomédicas Abel Salazar, Universidade do Porto, Porto, Portugal; Universidade Federal do Rio de Janeiro, Brazil

## Abstract

Tryparedoxins (TXNs) are oxidoreductases unique to trypanosomatids (including *Leishmania* and *Trypanosoma* parasites) that transfer reducing equivalents from trypanothione, the major thiol in these organisms, to sulfur-dependent peroxidases and other dithiol proteins. The existence of a TXN within the mitochondrion of trypanosomatids, capable of driving crucial redox pathways, is considered a requisite for normal parasite metabolism. Here this concept is shown not to apply to *Leishmania*. First, removal of the *Leishmania infantum* mitochondrial TXN (*LiTXN2*) by gene-targeting, had no significant effect on parasite survival, even in the context of an animal infection. Second, evidence is presented that no other TXN is capable of replacing *Li*TXN2. In fact, although a candidate substitute for *Li*TXN2 (*Li*TXN3) was found in the genome of *L. infantum*, this was shown in biochemical assays to be poorly reduced by trypanothione and to be unable to reduce sulfur-containing peroxidases. Definitive conclusion that *Li*TXN3 cannot directly reduce proteins located within inner mitochondrial compartments was provided by analysis of its subcellular localization and membrane topology, which revealed that *Li*TXN3 is a tail-anchored (TA) mitochondrial outer membrane protein presenting, as characteristic of TA proteins, its N-terminal end (containing the redox-active domain) exposed to the cytosol. This manuscript further proposes the separation of trypanosomatid *TXN* sequences into two classes and this is supported by phylogenetic analysis: i) class I, encoding active TXNs, and ii) class II, coding for TA proteins unlikely to function as TXNs. *Trypanosoma* possess only two TXNs, one belonging to class I (which is cytosolic) and the other to class II. Thus, as demonstrated for *Leishmania*, the mitochondrial redox metabolism in *Trypanosoma* may also be independent of TXN activity. The major implication of these findings is that mitochondrial functions previously thought to depend on the provision of electrons by a TXN enzyme must proceed differently.

## Introduction

Tryparedoxins (or TXNs) are oxidoreductases found exclusively in organisms of the family Trypanosomatidae, which includes medically relevant protozoan parasites of the genera *Leishmania* and *Trypanosoma*. TXNs are remotely related to enzymes of the thioredoxin family. When compared to these, TXNs share only 13% sequence identity, are larger in size (molecular weight of 16 kDa versus 11 kDa for thioredoxins) and exhibit a unique active site signature that reads Trp-Cys-Pro-Pro-Cys-Arg [instead of Trp–Cys–(Gly/Ala)–Pro–Cys–Lys for most thioredoxins]. Moreover, unlike thioredoxins, which are reduced enzymatically by thioredoxin reductase, TXNs are specifically reduced by trypanothione [T(SH)_2_], a conjugate of glutathione and spermidine that largely replaces glutathione functions in trypanosomatids and that is itself maintained in a reduced state by the flavoenzyme trypanothione reductase at the expense of NADPH. The reducing equivalents obtained from trypanothione are transferred by TXNs to a variety of thiol-containing molecules [1]. Accordingly, as thioredoxins, TXNs participate in different aspects of parasite development, many of which are crucial to life.

The first characterized activity of TXNs was the reduction of 2-Cys peroxiredoxins (2-Cys PRXs) [2], a ubiquitous class of sulfur-dependent peroxidases. For this reason, trypanosomatid 2-Cys PRXs were coined as tryparedoxin peroxidases or TXNPxs. The subsequent observation that TXNs are also the reductants for non-selenium glutathione peroxidase-like enzymes (nsGPXs) [3], [4], [5] reinforced the idea that TXNs are central players in trypanosomatid peroxide detoxification. Other TXN oxidants include ribonucleotide reductase [6], the universal minicircle sequence binding protein (UMSBP) [7] and a monothiol glutaredoxin (1-Cys GRX) [8], [9], *i.e.* molecules that are required for nuclear and mitochondrial DNA replication, and for iron-sulfur cluster biosynthesis, respectively.

TXNs can be found in the cytosol of all trypanosomatids, as is the case for the TXN1 molecules of *Leishmania infantum* [10], *Trypanosoma brucei* [11], *Trypanosoma cruzi* [3] and *Crithidia fasciculata* [12]. The importance of cytosolic TXNs for survival has been unequivocally demonstrated for *L. infantum* [13] and *T. brucei* [14], [15]. Since the mitochondria of trypanosomatids contain several enzymes that are specifically reduced by TXN, it is a common belief that these organisms also possess a mitochondrial TXN [1], [16], [17]. Still, the presence of a TXN within the mitochondrion has only been convincingly shown for the *L. infantum* TXN2 enzyme [10]. Such mitochondrial TXN is anticipated to be vital because many of these mitochondrial TXN-dependent molecules are essential to the parasite, namely mTXNPx (Castro H, Tomás AM, unpublished data), UMSBP [18], 1-Cys GRX [19] and possibly, as suggested by Schlecker *et al.* [20], nsGPX.

In this manuscript the actual need for an active mitochondrial TXN for normal parasite metabolism was investigated. For this, a *L. infantum* line unable to express the mitochondrial *Li*TXN2 enzyme was produced by gene targeting. The observation that these mutants behaved as wild type parasites throughout the entire *Leishmania* life cycle, raised the hypothesis that a second mitochondrial TXN, different from *Li*TXN2, would exist. To investigate this premise the genome of *L. infantum* was surveyed for additional *TXN* open reading frames (ORFs) and one candidate was found, *LiTXN3*. Biochemical characterization and subcellular localization studies of *Li*TXN3, nevertheless, revealed that this molecule was not capable of acting as a fully active TXN in the mitochondrion of *L. infantum*. All together, the data presented here demonstrates that, contrary to the accepted view, *Leishmania* can survive without an active TXN in their mitochondrion. Importantly, comparison of *TXN* sequences of *Leishmania* with those of *Trypanosoma*, along with an exhaustive phylogenetic analysis, strongly suggest that the non-requirement for TXN activity within the mitochondrion is also a feature of *Trypanosoma*. The consequences of this finding in what concerns the functioning of several mitochondrial processes are discussed.

## Results

### Generation of *LiTXN2* null mutants

**Figure 1:**
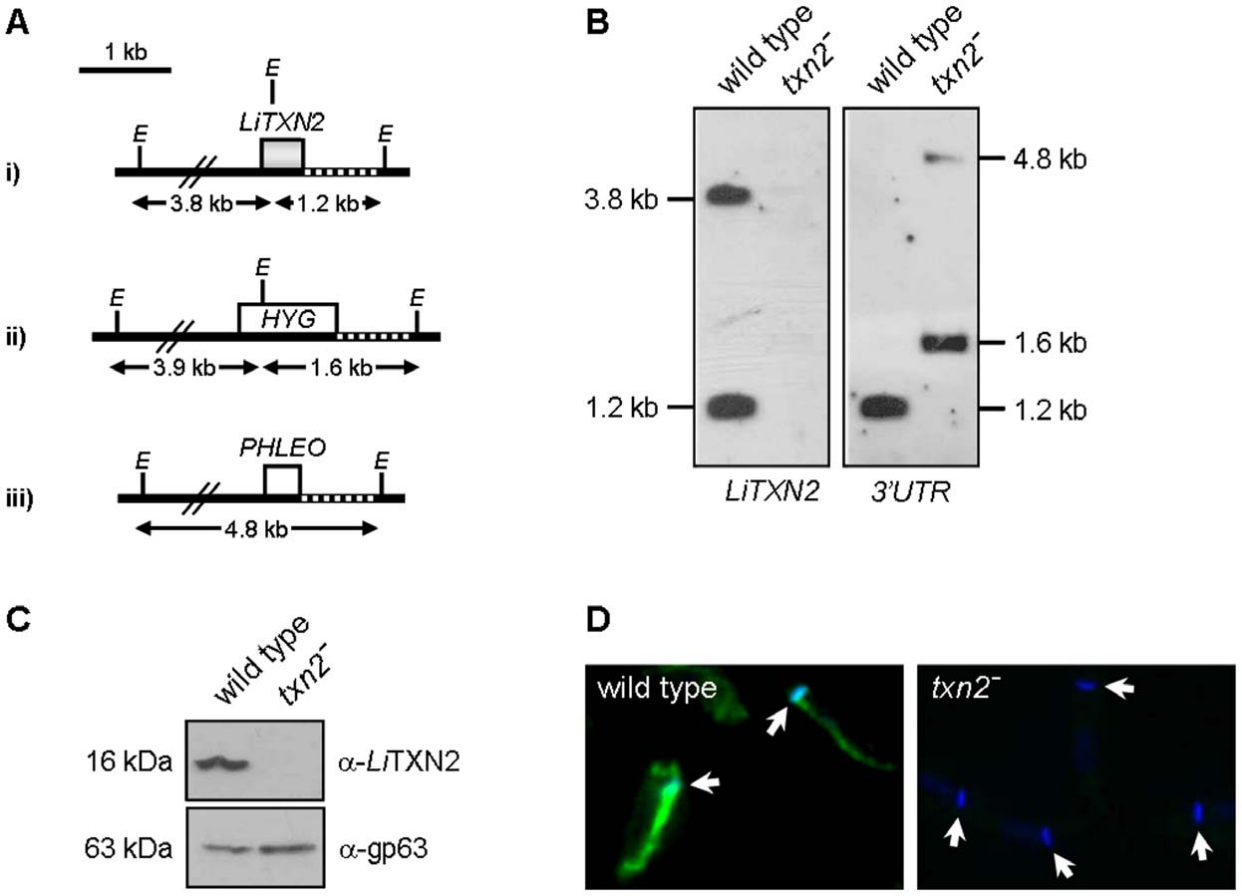
Gene-targeted deletion of *LiTXN2*. A. Map of the *LiTXN2 locus* i) before and after *LiTXN2* replacement by the ii) *HYG* and the iii) *PHLEO* constructs. Homologous recombination occurred via the 5′ and the 3′ flanking regions of the *LiTXN2* gene, cloned upstream and downstream of the selectable marker genes. Also depicted are the sizes of the *Eco*RI (*E*) fragments and the 3′-flanking region of *LiTXN2* (*3′UTR*, dashed line) used as probe in the Southern blot analysis. B. Southern blot analysis of *Eco*RI-digested genomic DNA of wild type parasites and homozygous Δ*txn2::HYG*/Δ*txn2::PHLEO* (*txn2^−^*) mutants, hybridized with the *LiTXN2* ORF and corresponding *3′UTR*. In *txn2^−^* mutants no band was detected when the *LiTXN2* ORF was used as probe. In these parasites the *3′UTR* probe labelled two bands of 1.6 kb and 4.8 kb, corresponding to the *HYG* and the *PHLEO* cassettes, respectively. C. Western blot analysis of 25 µg of total protein extracts from wild type and *txn2^−^* promastigotes incubated with the anti-*Li*TXN2 antibody. Upon stripping, the membrane was incubated with the anti-gp63 antibody, as control for loading. D. Analysis of wild type and *txn2^−^* promastigotes by indirect immunofluorescence. Parasites were fixed, permeabilized and incubated with the anti-*Li*TXN2 antibody (green labeling) and with DAPI (blue labeling). Arrows point to DAPI-stained mitochondrial DNA (kDNA).

To investigate the relevance of the mitochondrial *Li*TXN2 enzyme for *L. infantum* survival a parasite line unable to express this molecule was produced by homologous recombination. Since *Leishmania* have a diploid genome, two successive rounds of gene targeting were required to obtain *LiTXN2* homozygous knockout mutants. Accordingly, two linear DNA fragments containing either the *HYG* or the *PHLEO* genes, flanked by part of the upstream and downstream regions of *LiTXN2*, were introduced successively into parasites by electroporation (Figure 1A). Southern blot analysis of the resulting hygromycin- and phleomycin-resistant parasites confirmed the successful replacement of both *LiTXN2* alleles (Figure 1B). The double targeted mutants produced in this way (Δ*txn2::HYG*/Δ*txn2::PHLEO*, hereon referred to as *txn2^−^*) were further confirmed to lack *Li*TXN2 expression by western blot and indirect immunofluorescence analysis (Figure 1C–D).

### *Li*TXN2 is not essential throughout the parasite life cycle

**Figure 2:**
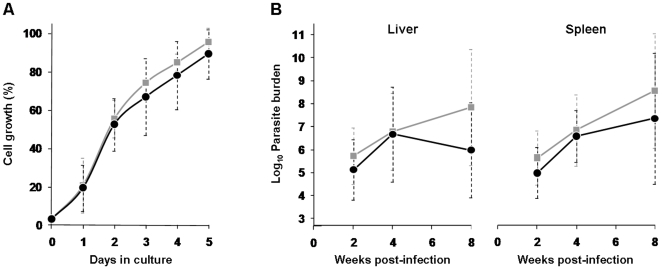
Depletion of *Li*TXN2 does not affect survival of *L. infantum* throughout its life cycle. A. Proliferation of promastigotes, either *txn2^−^* (black circles) or wild type (grey squares), monitored throughout 5 days of culture. Data are expressed as the percentage of parasite density relative to the highest value recorded at each experiment. Values represent mean and standard deviation of three independent growth curves. B. Survival of *L. infantum* intracellular amastigotes measured as the parasite burden in the livers (*left*) and spleens (*right*) of BALB/c mice. Parasite burdens were determined at different times after inoculation with either *txn2^−^* (black circles) or wild type (grey squares) parasites. No statistically significant differences were found between animals infected with *txn2^−^* and wild type parasites. Data represent mean and standard deviation of 2 independent experiments (involving a total of 21 animals infected with *txn2^−^*).

During its life cycle *Leishmania* proceed through two morphologically and physiologically distinct stages, the promastigote (an extracellular form residing in the insect vector) and the amastigote (an intracellular form that parasitises mammalian host macrophages). *LiTXN2* homozygous knockout mutants generated in the promastigote stage showed no obvious morphological alterations when compared to wild type parasites, not even in the kinetoplast (Figure 1D), that is, the structure that in trypanosomatids contains the mitochondrial DNA (kDNA). Moreover, under standard culture conditions, the growth rate of *txn2^−^* was similar to that of promastigotes expressing wild type levels of *Li*TXN2 (Figure 2A). These observations are particularly relevant because they argue against the participation of *Li*TXN2 in kDNA replication (via reduction of UMSBP), a function previously attributed to mitochondrial TXNs [7], [17].

To assess the consequences of *Li*TXN2 depletion on amastigote survival, *txn2^−^* mutants were inoculated into BALB/c mice, an animal model for *Leishmania* infection. Since *L. infantum* is an agent of visceral leishmaniasis, at defined time points after infection, parasite burdens were evaluated in the liver and spleen of infected mice by the limiting dilution assay. As shown in Figure 2B, elimination of *Li*TXN2 did not affect the ability of *L. infantum* to replicate and give rise to a productive infection in a mammalian host. Indeed, the parasitemia produced by *txn2^−^* was not significantly different from that observed with wild type parasites. The demonstration that *Li*TXN2 is not essential throughout the *L. infantum* life cycle suggested that, in addition to *Li*TXN2, a second TXN would be active within the *L. infantum* mitochondrion.

### *Li*TXN1 is restricted to the cytosol

**Figure 3:**
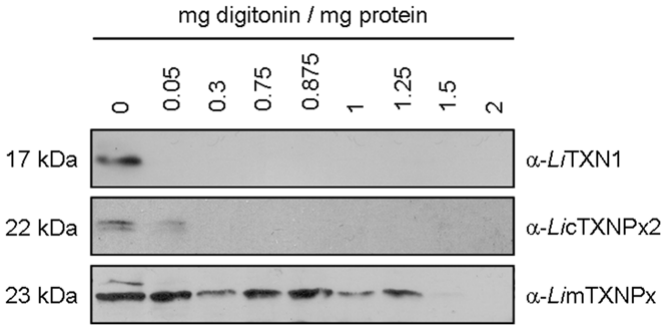
The cytosolic *Li*TXN1 enzyme is not present in the mitochondrion of *txn2^−^* promastigotes. Protein samples resulting from the permeabilization of *txn2*ù promastigotes with increasing concentrations of digitonin in the presence of proteinase K were analyzed by western blot using anti-*Li*TXN1 sera. Anti-*Li*cTXNPx2 and anti-*Li*mTXNPx were employed to detect cytosolic and mitochondrial compartments, respectively.

In biochemical assays mitochondrial thiol-containing molecules, such as *Li*mTXNPx, can be reduced by the cytosolic enzyme *Li*TXN1 [10]. It is nevertheless unlikely that *Li*TXN1 interacts physiologically with such proteins owing to their distinct subcellular compartmentalization. Using parasites lacking *Li*TXN2 expression and a digitonin/proteinase K assay, *Li*TXN1 was confirmed to be absent from the mitochondrion. Based on the differential sterol content of their membranes, the various subcellular compartments can be selectively permeabilized by digitonin, the mitochondrial membrane being more resistant to this detergent than the cell membrane. When proteinase K (PK) and digitonin are added to the cells simultaneously, PK will only gain access to, and hence digest, the protein content of permeated organelles. Western blot analysis of samples resulting from treatment of *txn2^−^* mutants with digitonin/PK (Figure 3) showed that *Li*TXN1 is readily digested at digitonin concentrations that are too low to permeabilize mitochondrial membranes (controlled by the anti-*Li*mTXNPx antibody). In other words, and in agreement with bioinformatic predictions (MitoProtII, Predotar, PlasMit), *Li*TXN1 is not present in the mitochondrion of *txn2^−^* parasites. Therefore, if *L. infantum* does harbor a second TXN in its mitochondrion this is not *Li*TXN1.

### Screening of the *L. infantum* genome uncovers *LiTXN3*, a candidate substitute for *LiTXN2*

To investigate the existence of alternative mitochondrial TXNs the *L. infantum* genome was screened for *TXN* coding sequences and found to contain, apart from the well characterized *LiTXN2* and *LiTXN1* genes, five additional sequences, designated here as *LiTXN3*, *LiTXN4*, *LiTXN5*, *LiTXN6* and *LiTXN7* (Gene IDs: LinJ31_V3.2000, LinJ31_V3.2010, LinJ29_V3.1230, LinJ29_V3.1220 and LinJ35_V3.0380, respectively, according to the TriTrypDB). However, with the exception of *LiTXN3*, all these ORFs encode theoretical proteins with amino acid substitutions that preclude them from acting as TXNs (for additional information refer to Text S1 and Figure S1). These include alterations in the TXN active site motif, as well as in the residues that form the hydrogen bond network required for normal TXN activity [21]. Accordingly, only *LiTXN3* can be regarded as a candidate substitute for *LiTXN2* and, as such, its characterization was pursued here.

**Figure 4:**
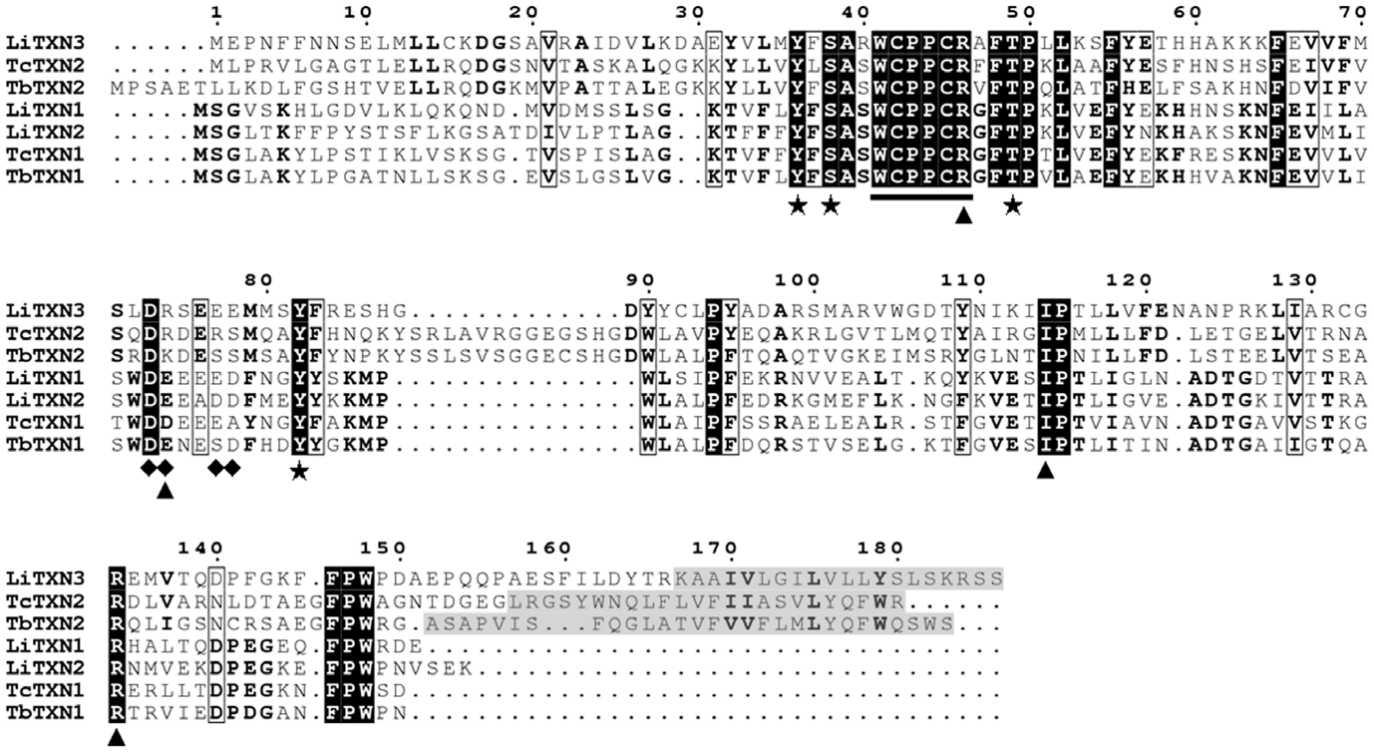
Sequence alignment of *Li*TXN3 with other TXNs. The first three sequences in the alignment refer to the TXN3 enzyme of *L. infantum* (*Li*) and to the TXN2 molecules of both *T. cruzi* (*Tc*) and *T. brucei* (*Tb*), all of which possess C-terminal hydrophobic tails (marked on light grey background), predicted to be transmembrane domains (by bioinformatics analysis using TMpred and TopPred). The remaining four sequences are molecules characterized previously and known to display classical TXN activity. Strict identity across all sequences is represented with white letters on a black background. These include the TXNs active site signature (highlighted with a bar). Similarity across both subgroups is marked with black frames. Residue similarity in each subgroup is indicated with bold letters. Residues involved in the hydrogen bond system are marked with stars. Residues implicated in reaction with trypanothione are marked with arrowheads (Arg134 is also likely involved in binding to 2-Cys PRXs). The acidic area, important for interaction with 2-Cys PRXs, is marked with diamonds. Sequence numbering refers to *Li*TXN3. Gene IDs attributed by TriTrypDB (May 2010) are as follows: *Li*TXN3, LinJ31_V3.2000; *Tc*TXN2, Tc00.1047053509997.20; *Tb*TXN2, Tb927.3.5090; *Li*TXN1, LinJ29_V3.1250; *Li*TXN2, LinJ29_V3.1240; TcTXN1; Tc00.1047053509997.30; *Tb*TXN1, Tb927.3.3780.

*LiTXN3* encodes a protein with a theoretical molecular weight of 21.6 kDa and a pI of 6.82. Figure 4 shows the *Li*TXN3 predicted amino acid sequence and compares it to TXNs known to display activity (*Li*TXN1, *Li*TXN2, *Tb*TXN1 and *Tc*TXN1) [3], [10], [11], as well as to its counterparts in *T. brucei and T. cruzi* (*Tb*TXN2 and *Tc*TXN2, respectively). An overview of this alignment reveals three major alterations in the amino acidic sequence of *Li*TXN3 relative to formerly characterized TXNs. First, at position 74 (*Li*TXN3 numbering), where classical enzymes harbor a Glu or an Asp, *Li*TXN3 contains a basic Arg residue. An acidic residue at this position was described to be important for TXN interaction with trypanothione [21], as well as with 2-Cys PRXs [22]. Accordingly, when the negative charge at position 74 was reversed in *Cf*TXN2 and *Tb*TXN1, the specific activities of these proteins dropped to 17% and 41% of the wild-type levels, respectively [21], [22]. Second, position 40 in *Li*TXN3 is occupied by an Arg residue, replacing the Ser present in other TXNs. This Ser is in the vicinity of an acidic area proposed to be important for the contact between TXN and 2-Cys PRXs [22]. Finally, *Li*TXN3 has a Lys at position 112, instead of a Glu residue, reported to be involved in interaction of TXNs with nsGPXs [23], the second family of TXN-dependent peroxidases. Of notice, with the exception of Arg 40, the predicted TXN2 sequences of trypanosomes also present alterations in the positions notified above for *Li*TXN3 (Figure 4).

In addition to the modifications referred to above, there is one sequence feature that readily distinguishes *Li*TXN3 from all TXN enzymes characterized to date, which is the presence of a C-terminus extension enriched in hydrophobic amino acids. Based on bioinformatics analysis (using TopPred and TMpred) this hydrophobic C-tail is predicted to specify a transmembrane domain. Again, such feature is maintained in the TXN2 enzymes of *Trypanosoma* (Figure 4).

### *Li*TXN3 retains oxidoreductase but not TXN activity

To investigate whether *Li*TXN3 displays TXN activity, the enzyme was expressed in bacteria and purified as an N-terminal His-tagged recombinant protein, however most of it aggregated as inclusion bodies and the yield of soluble enzyme was very poor (data not shown). To overcome this limitation and to maximize the chances of obtaining correctly folded and active *Li*TXN3, a truncated version of the protein lacking the C-terminal hydrophobic sequence (6His*Li*ΔTXN3) was produced. Unless otherwise indicated all enzymatic assays were performed with the truncated enzyme, using recombinant *Li*TXN2 [10] as a positive control for TXN activity.

**Figure 5:**
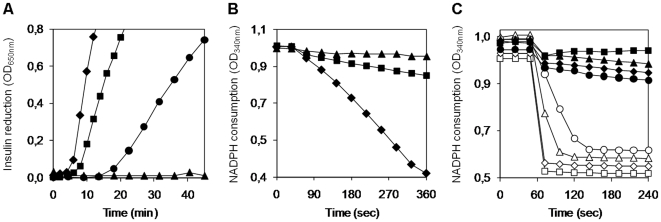
*Li*TXN3 displays oxidoreductase but not tryparedoxin activity. A. Formation of the insoluble insulin B chain (monitored at 650 nm) by DTT-reduced *Li*ΔTXN3 (squares) or *Li*TXN2 (diamonds), or by *Li*ΔTXN3 reduced in the presence of NADPH, TR and T(SH)_2_ (circles). Negative controls were performed in the same reaction mixtures [either with DTT or with the TR/T(SH)_2_ redox system] without any addition of TXN (triangles). B. Reduction of *Li*ΔTXN3 by T(SH)_2_ followed by monitoring NADPH consumption at 340 nm. Reaction systems contained NADPH, TR and T(SH)_2_ as TXN reductant and insulin as final electron acceptor. The TXNs tested were *Li*ΔTXN3 (squares) and *Li*TXN2 (diamonds). Negative control contains no TXN (triangles). C. Reduction of TXN-dependent peroxidases by *Li*ΔTXN3. Reaction mixtures consisted in NADPH, TR, T(SH)_2_, recombinant TXN [either *Li*ΔTXN3 (closed symbols) or *Li*TXN2 (open symbols)] and four different peroxidases, namely *Li*mTXNPx (triangles), *Li*cTXNPx1 (squares), *Li*cTXNPx2 (diamonds) and *Li*nsGPXA1 (circles). Reactions were initiated by addition of H_2_O_2_ and NADPH consumption was followed at 340 nm.

In the classical Holmgren assay [24], 6His*Li*ΔTXN3 proved to be an active oxidoreductase. Indeed, when incubated with DTT, *Li*ΔTXN3 was capable of reducing insulin (Figure 5A), a reaction that results in aggregation of the insoluble insulin B chain and that can be monitored spectrophotometrically at 650 nm. When DTT was replaced by trypanothione, *Li*ΔTXN3 could still reduce insulin, albeit at a lower rate (Figure 5A), whereas dihydrolipoamide, another mitochondrial reductant, could only marginally support the activity of *Li*ΔTXN3 on insulin (data not shown). Neither thioredoxin nor glutathione served as reductants for *Li*ΔTXN3 in this assay (data not shown).

To get an insight into the efficiency of trypanothione catalyzed *Li*ΔTXN3 reduction, NADPH consumption was monitored (at 340 nm) in the presence of trypanothione reductase (TR), trypanothione, recombinant TXN and insulin (Figure 5B). Since reduction of TXN by trypanothione is the limiting step in this cascade [10], the slopes of NADPH consumption reflect the velocity of this reaction. As depicted in Figure 5B, when compared with *Li*TXN2, *Li*ΔTXN3 was found to be poorly reduced by trypanothione.

Finally, when tested in the routine assay for reduction of TXN-dependent peroxidases [2] *Li*ΔTXN3 exhibited negligible activity (Figure 5C). The peroxidases assayed were the mitochondrial 2-Cys PRX (*Li*mTXNPx) [25] and two of its cytosolic isoforms (*Li*cTXNPx1 and *Li*cTXNPx2) [10], as well as a nsGPX (*Li*nsGPxA1; Gene ID LinJ26_V3.0770). The full-length 6His*Li*TXN3 was also tested in this assay towards *Li*cTXNPx1, *Li*cTXNPx2 and *Li*mTXNPx, with the same results (data not shown).

In short, by showing that *Li*TXN3 was unable to reduce TXN-dependent peroxidases these results confirm what the amino acid substitutions in the *Li*TXN3 sequence (Figure 4) suggested, that this enzyme does not function as a classical TXN. Also in accordance with primary structure predictions, the reaction between *Li*TXN3 and trypanothione is considerably impaired but not hindered. Evidence is also provided that *Li*TXN3 retains oxidoreductase activity, making it reasonable to speculate that, although unable to reduce 2-Cys PRXs and nsGPXs, it could still sustain the activity of mitochondrial enzymes such as UMSBP and 1-Cys GRX. For this, however, *Li*TXN3 would have to share with these enzymes the same subcellular localization.

### *Li*TXN3 localizes to the mitochondrion

**Figure 6:**
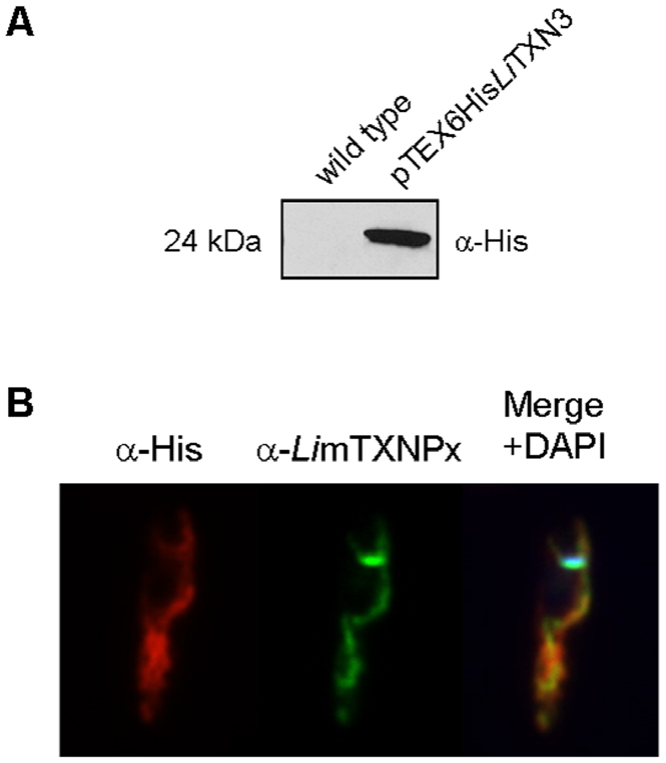
*Li*TXN3 localizes to the mitochondrion. A. Western blot analysis of 50 µg of total protein extract from wild type *L. infantum* and from promastigotes carrying the pTEX6His*Li*TXN3 episome (see Material and Methods). The blot was hybridized with anti-His to detect the 6His*Li*TXN3 chimera expressed by the transfected cell line. B. Immunolocalization of the 6His*Li*TXN3 chimera in pTEX6His*Li*TXN3-transformed promastigotes using the anti-His antibody (in red). The control mitochondrial *Li*mTXNPx enzyme was detected with an antibody produced against this protein (marked in green). Also included is the merging of both channels plus the channel for DNA staining with DAPI.

To address the subcellular localization of *Li*TXN3, immunolocalization studies were carried out using a transgenic *L. infantum* line expressing *Li*TXN3 as an N-terminal His-tagged chimera (see Materials and Methods) and subsequent detection with a specific anti-histidine antibody (Figure 6A). Immunofluorescence analysis of 6His*Li*TXN3-expressing parasites revealed that the subcellular distribution of this protein was similar to that of the mitochondrial enzyme *Li*mTXNPx [26] (Figure 6B). However, these two enzymes did not entirely co-localize, looking as if *Li*TXN3 contours *Li*mTXNPx. This might be due to the fact that *Li*mTXNPx is a soluble enzyme, whereas *Li*TXN3 is predicted to be a membrane protein.

### *Li*TXN3 is an integral membrane protein with its redox-active domain exposed to the cytosol

**Figure 7:**
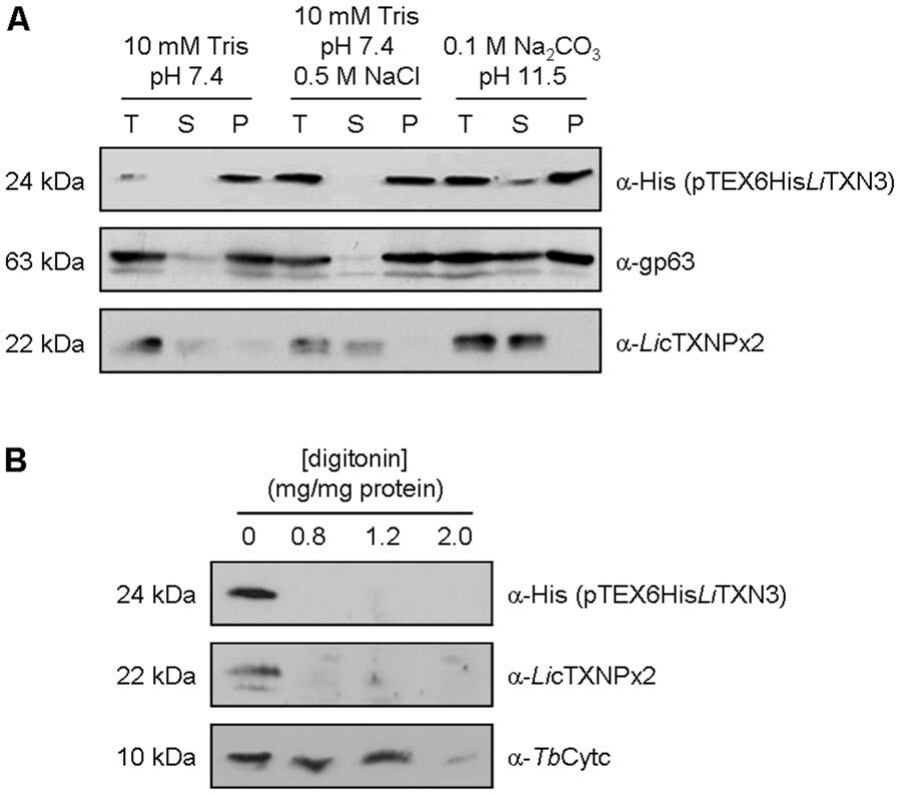
*Li*TXN3 is an integral membrane protein with the N-terminal domain exposed to the cytosol. A. Alkaline carbonate extraction performed on 6His*Li*TXN3-expressing *L. infantum* promastigotes. Parasites were disrupted in either 10 mM Tris-HCl pH 7.4, 10 mM Tris-HCl 0.5 M NaCl pH 7.4, or 0.1 M Na_2_CO_3_ pH 11.5 and fractionated by ultracentrifugation. Total protein extracts (T), as well as supernatants (S) and membrane pellets (P) resulting from fractionation were analyzed by western blot using anti-His antibody to detect 6His*Li*TXN3. Anti-gp63 and anti-*Li*cTXNPx2 antibodies were used to control for membrane and hydrophilic proteins, respectively. B. Protein samples resulting from permeabilization of 6His*Li*TXN3-expressing promastigotes with increasing concentrations of digitonin in the presence of proteinase K were analyzed by western blot using an anti-His antibody. Other antibodies were used to detect the cytosolic protein *Li*cTXNPx2, as well as a mitochondrial intermembrane space molecule (cytochrome c, detected with anti-*Tb*Cytc).

The confirmation that *Li*TXN3 is a membrane-bound protein was achieved by performing an alkaline carbonate extraction on 6His*Li*TXN3-expressing parasites. The results in Figure 7A show that *Li*TXN3 was recovered in the insoluble fractions of parasite lysates prepared in low and high salt solutions, and only partially solubilized with the alkaline carbonate buffer, an elution profile that matches that of the membrane protein gp63, but not with that of a soluble molecule (*Li*cTXNPx2).

The association of *Li*TXN3 with cell membranes must occur through its single C-terminal stretch of hydrophobic residues, rendering it a C-tail anchored (TA) protein [27]. Mitochondrial TA proteins are always attached to the outer membrane and expose their functional N-terminal domains to the cytosol [27]. This was confirmed to be also the case with *Li*TXN3 by employing a digitonin/PK assay. As shown in Figure 7B, the N-terminal portion of *Li*TXN3 (detected with the anti-His antibody) was digested at digitonin concentrations that expose cytosolic proteins to PK (at 0.8 mg digitonin/mg protein; controlled with the anti-*Li*cTXNPx2 antibody), but that do not enable PK to reach the mitochondrial compartments. Access of PK to the mitochondrion could be tracked using an antibody specific for cytochrome c, a protein of the mitochondrial intermembrane space [28].

Altogether, this set of experiments demonstrates that *Li*TXN3 is an outer mitochondrial protein with its hydrophilic portion (containing the TXN active site) facing the cytosol. In other words, *Li*TXN3 cannot directly react with molecules that are located within the mitochondrial compartments, namely mTXNPx, nsGPX and UMSBP, and therefore sustain the biological processes driven by these proteins.

### Phylogenetic analysis of TXNs does not support the existence of a mitochondrial TXN in *Trypanosoma*

**Figure 8:**
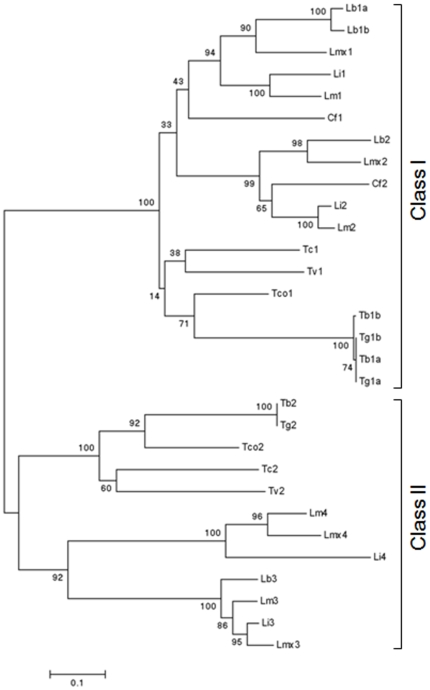
Phylogenetic analysis of *TXN* genes of trypanosomatids showing their separation into two distinct classes. Neighbor-joining tree with bootstrapped values (500 replicates) Cf: *Crithidia fasciculata*; Lb: *L. brazilienses*; Li: *L. infantum*; Lm: *L. major*; Lmx: *L. mexicana*; Tb: *T. brucei brucei*; Tg: *T. brucei gambienese*; Tc: *T. cruzi*; Tco: *T. congolense*; Tv: *T. vivax*.

*Li*TXN3 is a protein with sequence features that are not present in any of the active TXNs described to date (*i.e.* the cytosolic TXN1 enzymes and the mitochondrial TXN2 of *L. infantum*), but are maintained in the TXN2 enzymes of *Trypanosoma*. To gain insight into how these different TXNs relate to previously characterized enzymes at an evolutionary level, a phylogenetic analysis was performed using 30 DNA sequences, representative of 9 species of the genera *Leishmania*, *Trypanosoma* and *Crithidia*. Using the MEGA4 software [29], several methods were employed to construct various phylogenetic trees all of which, despite discrete differences, conserved the main features. The topology of one representative tree is shown in Figure 8. Remarkably, the 30 *TXN* ORFs analyzed separate into two distinct evolutionary branches which reflect the differences observed at the biochemical level and can, thus, be regarded as two different classes. The first of these classes contains the genes encoding the cytosolic TXNs of all trypanosomatids (*TXN1*), as well as the mitochondrial TXNs of *Leishmania* (*TXN2*). The second class, in which *Leishmania TXN3* and *Trypanosoma TXN2* are included, contains only sequences encoding proteins with a C-terminal hydrophobic extension typical of TA proteins and possessing alterations in amino acids required for classical TXN activity. The presence of members of these two classes in all trypanosomatids indicates that they were already contained within the trypanosomatid ancestor. *Trypanosoma cruzi*, maintains what is possibly the primitive situation, *i.e.* a single representative of each class in the same genomic *locus*, while *T. brucei*, for instance, have recently duplicated the *TXN1* ORF and *Leishmania* have undergone further genetic rearrangements, such that, presently, they contain more than one member in each class (*TXN1* and *TXN2* in class I, *TXN3* and *TXN4* in class II). Noticeably, the tree represented in Fig. 8 further shows that the *TXN2* of *Leishmania* (encoding the mitochondrial enzymes) and the *TXN2* of *Crithidia* form a clear subgroup within class I whose origin must have preceded *Leishmania* and *Crithidia* speciation. Additionally, the tight definition of this subgroup (bootstrap support of 99%) and the fact that it contains no *Trypanosoma* sequences, strongly suggest that its precursor gene arose after *Trypanosoma* divergence or has been lost by the predecessor of this genus.

In short, phylogenetic analysis of TXN sequences provides i) an explanation for the presence of fully active mitochondrial TXNs in *Leishmania* (*e.g. Li*TXN2), but not in *Trypanosoma*, and ii) demonstrates that *Leishmania TXN3* genes group together with *Trypanosoma TXN2* sequences in a separate class of TXNs whose distinctive feature is the presence of a C-terminal hydrophobic tail. The function of this class of TXNs is yet to be determined but characterization of the *Li*TXN3 member strongly suggests that these enzymes do not act as inner mitochondrial TXNs.

## Discussion

In the cytosol of trypanosomatid parasites (*e.g. L. infantum*, *T. brucei* and *T. cruzi*) TXNs enzymes transfer reducing equivalents from trypanothione to redox pathways involving thiol/disulfide exchange. Several TXN-dependent enzymes have also been identified within the mitochondrion of different trypanosomatids and so it is generally accepted that likewise a fully active TXN must also be present within the mitochondrion of these organisms. Such TXN would fuel essential pathways such as peroxide detoxification and mitochondrial DNA replication, and could also participate in iron-sulfur cluster biosynthesis [1]. The actual need for an active TXN operating within the mitochondrion was challenged in this report, by demonstrating that the mitochondrial *Li*TXN2 enzyme of *L. infantum* is not vital for parasite viability or successful infectivity. Moreover, evidence was provided that no other *L. infantum* TXN was capable of replacing *Li*TXN2 functions. Importantly, as discussed next, the non-requirement for an inner mitochondrial TXN should not be exclusive of *Leishmania*, but instead, a feature common to all trypanosomatids.

The *Trypanosoma* genomes contain, apart from *TXN1* (that codes for the cytosolic TXN1 enzyme), an extra TXN coding sequence, *TXN2*. It is generally accepted that *Trypanosoma TXN2* genes encode *the* mitochondrial TXN of these organisms [1], [16], [17]. Still, the only evidence for this is a report where overexpression of the *T. brucei Tb*TXN2 reversed a defective phenotype induced by excess of mitochondrial enzymes involved in redox reactions, either a cytochrome b5 reductase-like protein (CBRL) or the mitochondrial TXN-dependent peroxidase mTXNPx [17], even though no such defect was evident when mTXNPx was overexpressed in *T. cruzi* [30] and *Leishmania* [26], [31], [32]. The data presented in this manuscript raises serious doubts regarding the participation of *Trypanosoma* TXN2 enzymes in intramitochondrial redox processes. Phylogenetic analysis shows that *Trypanosoma TXN2* and *Leishmania TXN2* sequences, despite sharing the same nomenclature, are not orthologues. Instead, *Trypanosoma TXN2* genes cluster together with *Leishmania TXN3* in a class of *TXNs* (class II) that is distinct from the one containing the *TXN1* of all trypanosomatids and the *TXN2* sequence of *Leishmania* and *Crithidia* (class I). Contrary to class I members, class II TXNs are unlikely to function as TXNs owing to the presence of a number of non-identical amino acid substitutions. Although direct evidence for this was only obtained for *Li*TXN3, *Trypanosoma* TXN2 share with *Li*TXN3 all sequence alterations likely to impair TXN activity, the exception being a Ser residue at position 40 (*Li*TXN3 numbering), preserved in *Trypanosoma* TXN2, but replaced by an Arg in *Li*TXN3. By showing that this amino acid substitution is not responsible for the defective activity of *Li*TXN3 (see Text S2 and Figure S2), the hypothesis that *Trypanosoma* TXN2 enzymes behave differently from *Li*TXN3, *i.e.* that they retain TXN activity, has no sustainability. An additional reason precluding class II TXNs from reducing molecules present in the mitochondrial compartments is their subcellular compartmentalization. All class II TXNs possess a single C-terminal hydrophobic domain that is a defining feature of TA proteins [27]. The transmembrane domain of TA proteins determines their fate in the cell by directing and anchoring them either on the mitochondrial outer membrane, or on the membranes of the ER or of the peroxisomes (which in trypanosomatids would be the glycosomes), while leaving the entire functional N-terminal portion (which in TXNs contains the redox active domain) facing the cytosol. The subcellular localization of *Li*TXN3, along with its membrane topology, leave no doubts that this TXN is a TA protein (more specifically, anchored on the mitochondrial outer membrane). Again, although *Trypanosoma* TXN2 were not submitted to experimental scrutiny, comparison of these sequences with that of *Li*TXN3 strongly suggests that they are also TA members and, as such, expose their redox-active domain towards the cytosol. Since the *Trypanosoma* genomes do not harbor any other gene capable of coding for a fully active mitochondrial TXN, the concept that a mitochondrial TXN is not required for normal cell metabolism, unequivocally demonstrated here for *Leishmania*, can be extended to *Trypanosoma*.

The proposal that no mitochondrial TXN is essential for trypanosomatid viability implies that several redox pathways that take place in this organelle cannot proceed as currently thought. Rather, an alternative reducing agent(s) in the form of a different thiol-containing protein or of low molecular mass thiol, is required to reduce, among other components, mTXNPx, nsGPX, UMSBP and 1-Cys GRX, that is, enzymes involved in processes as vital as peroxide metabolism, kinetoplast replication and iron sulfur cluster biosynthesis, respectively [1]. The observation that both *E. coli* and *T. brucei* thioredoxin reduces TXNPxs [25], [33], 1-Cys GRX [8] and nsGPX [4], albeit much less efficiently than does TXN, would support thioredoxin as a candidate for TXN substitution if these are to be shown allocated to the mitochondrion. Moreover, and even if proposing an entity for a TXN alternative is not straightforward, the genome of trypanosomatids encodes several other putative dithiol-containing proteins whose role as mitochondrial oxidoreductases is worth investigating. In an equally conceivable hypothesis the trypanosomatids' major thiol, trypanothione, could deliver electrons directly to mTXNPxs, nsGPXs, UMSBP, and 1-Cys GRX among other proteins, overcoming the need for an intermediate oxidoreductase. However, biochemical assays only showed trypanothione-driven reduction in the case of the *T. brucei* 1-Cys GRX [8]. Neither the peroxidases mTXNPx [25] and nsGPX [4] nor UMSBP [34], the enzyme that initiates replication of the kinetoplast, were found to receive electrons directly from this thiol. Trypanothione might also provide a simple explanation for elimination of peroxides produced within mitochondria in the absence of an active TXN. In fact, at millimolar concentrations, trypanothione scavenges peroxides, such as hydrogen peroxide and peroxynitrite, directly and fast enough to account for effective detoxification [4], [35], [36]. Therefore, if such concentrations are reached in mitochondria, peroxide detoxification in this organelle could bypass the requirement for peroxidase-catalyzed reactions. Furthermore, other low molecular mass thiols (*e.g.* glutathione, ovothiol or free cysteine), as well as thiols exposed on protein surfaces [37] and, in the case of *Leishmania*, an intermembrane space located ascorbate peroxidase [38] might assist trypanothione in mitochondrial antioxidant defense. Accurate determination of the concentration of trypanothione and of other thiols in the mitochondria of the several trypanosomatids will certainly shed light on the likelihood that non-protein thiols directly reduce peroxides as well as enzymes currently considered as TXN targets. In this regard, a comparative analysis of antioxidant molecules between *L. infantum* lines with and without *Li*TXN2 may also provide relevant information.

Apart from showing that trypanosomatids can thrive in the absence of an active TXN in their mitochondria, this work is novel in characterizing a member (*Li*TXN3) of a new class of TXNs, unique for being TA. Tail anchored proteins embrace a group of transmembrane molecules, which are increasingly recognized as critical for the normal functioning of the different organelles that harbor them. Some of these proteins include components of the translocation machinery of the mitochondria [39], as well as mitochondrial outer membrane proteins that regulate apoptosis [40] and mitochondrial morphology [41]. In the particular case of *Li*TXN3, the finding that this is an outer mitochondrial protein with moderate oxidoreductase activity could indicate that it is involved in redox-dependent biological processes. By analogy with thioredoxins, such redox processes could include protein folding assistance, protease activation and intracellular signaling [42], [43]. In this sense, further characterization of *Li*TXN3 and their counterparts in *Trypanosoma* spp. is important to address if such oxidoreductase function exists in the context of the cell. Nevertheless, the possibility that the function of class II TXNs members is independent of their redox activity cannot be excluded in a similar way to what has been previously described to a eukaryote thioredoxin [44]. In either case the essential character of class II TXNs and their amenability to specific inactivation are worth investigating.

In summary, by demonstrating that trypanosomatids do not require an active tryparedoxin within their mitochondrion, the experimental work contained in this paper establishes that the redox metabolism in this organelle is not dependent on the activity of this family of enzymes. This implies reformulating the model in which a central TXN molecule supplies reducing equivalents to critical mitochondrial sulphur-containing enzymes, such as those involved in peroxide elimination and kinetoplast replication. From a different perspective, this manuscript characterized for the first time a member of a new class of TXNs that does not display classical TXN activity and whose physiological function is as yet uncovered. The chapter on tryparedoxin systems is definitely not closed.

## Materials and Methods

### Ethics statement

The experimental animal procedures were approved by the Local Animal Ethics Committee of Institute for Molecular and Cell Biology, University of Porto, Portugal and licensed by DGV (General Directory of Veterinary, Ministry of Agriculture, Rural Development and Fishing, Govt. of Portugal), in May 18, 2006 with reference 520/000/000/2006. All animals were handled in strict accordance with good animal practice as defined by national authorities (DGV, Law n°1005/92 from 23^rd^ October) and European legislation EEC/86/609.

### Parasite culture and genetic manipulations

Wild type and transfected *Leishmania infantum* promastigostes (MHOM MA67ITMAP263) were cultured at 25°C in RPMI 1640 with GlutaMAX, (Invitrogen) supplemented with 10% inactivated fetal bovine serum (FBSi, Invitrogen), 20 mM hepes sodium salt (pH 7.4) and 50 U ml^−1^ penicillin, 50 µg ml^−1^ streptomycin. For growth rate determination *L. infantum* promastigotes previously synchronized by 4–5 daily passages at 5×10^5^ cells ml^−1^, were cultured at 10^6^ cells ml^−1^ and the cell density monitored over time with a hematocytometer. Transfection of promastigotes was performed as described elsewhere [45]. Isolated clones were picked from agar plates containing selective drugs and transferred into liquid medium. Geneticin (G418; Sigma) was used at 15 µg ml^−1^, hygromycin (Invitrogen) at 10 µg ml^−1^ and phleomycin (Sigma) at 17.5 µg ml^−1^.

### Construction of the *LiTXN2* replacement vectors

To assemble the *HYG* and the *PHLEO* replacement vectors, the 5′ and 3′ non-coding sequences flanking the *LiTXN2* open reading frame were PCR amplified from *L. infantum* genomic DNA using the primers 5′-cccaagcttTGCTCGGCATCTACACC-3′, 5′-cggactagtGGTAGCAAAGATGTCAGG-3′, 5′-cgcggatccACCAAACACAAAGGGGTG-3′ and 5′-cgcgagctcGCACCGCACAATATACGG-3′, which incorporate the *Hind*III, *Spe*I, *Bam*HI and *Sac*I restriction sites (clamp sequences in lower case; restriction sites underlined), respectively. Following digestion with the appropriate restriction enzymes, the PCR products were cloned into the *Hind*III-*Spe*I and *Bam*HI-*Sac*I sites of the *HYG* and *PHLEO* constructs used previously to target the *LiTXN1* ORF [13], on both sides of the hygromycin- and phleomycin-resistance gene (*HYG* and *PHLEO*, respectively). Previous to transfection both constructs were linearized with *Hind*III and *Sac*I restriction enzymes and purified from agarose gels by electroelution.

### Construction of the 6His*Li*TXN3 expression vector

For construction of pTEX6His*Li*TXN3 the *LiTXN3* ORF was PCR amplified with PWO polymerase (Roche) using the primers 5′-cgcgcacatATGGAGCCCAACTTCTTCAAC-3′ and 5′-caccgctcgagTCACAAGCTGCGGCTGAT-3′ (clamp sequences in lower case; restriction sites underlined) and cloned into the *Nde*I-*Xho*I restriction sites of pET28a (Novagen). The 6His*Li*TXN3 chimera (with an N-terminal 6-His tag) was then excised from pET28a with *Bgl*II/*Xho*I and cloned into the corresponding sites of pTEX [46].

### Determination of parasitemia indexes by the limiting dilution assay

BALB/c and National Marine Research Institute (NMRI) mice were purchased from Charles River (Lyon, France). Mice were raised in specific pathogen-free conditions at the animal house facility of the Institute for Molecular and Cell Biology (IBMC). Prior to the infection experiments, all promastigote lines were passaged in NMRI mice. Next, 10^8^ *L. infantum* stationary phase promastigotes, were inoculated intraperitoneally into 6 to 9 weeks old male BALB/c mice. At defined time points after infection, mice were sacrificed and their livers and spleens excised, weighed and homogenized in Schneider's medium (Sigma) supplemented with 10% FBSi, 5 mM hepes sodium salt (pH 7.4), 100 U ml^−1^ penicillin, 100 µg ml^−1^ streptomycin, 5 µg ml^−1^ phenol-red and 2% sterile human urine. Homogenates were then diluted to 10 mg ml^−1^ and these cell suspensions titrated across a 96-well plate in serial four-fold dilutions (four titrations per organ) in quadruplicate. After two weeks of growth at 25°C, the last dilution containing promastigotes was recorded and the number of parasites per gram of organ (parasite burden) calculated as described elsewhere [47].

### Indirect immunofluorescence assay

Immunofluorescence assays were performed according to Castro *et al.* [26]. Briefly, recombinant parasites were fixed with 4% paraformaldehyde (w/v) in 0.1 M Na_2_HPO_4_, 0.1 M NaH_2_PO_4_, 0.15 M NaCl pH 7.2 (PBS), permeabilized with 0.1% (v/v) Triton X-100 (prepared in PBS), spotted onto polylysine-coated microscope slides and incubated with appropriate antibodies. Primary antibodies were anti-*Li*TXN2 [10], anti-*Li*mTXNPx [26] and anti-His (Abcam). Secondary antibodies were Alexa Fluor 488 anti-mouse IgGs, and Alexa Fluor 488 and 568 anti-rabbit (Molecular Probes). Slides were mounted in Vectashield (Vector Laboratories) and examined with an AxioImager Z1 microscope, equipped with an Axiocam MR ver.3.0 camera and the Axiovision 4.7 software (all from Carl Zeiss).

### Digitonin/proteinase K assay

Twelve and a half million promastigotes (about 25 µg of total protein) suspended in 18.75 µl DIG buffer (25 mM Tris, pH 7.5, 1 mM EDTA, 0.6 M sucrose) containing 50 µg ml^−1^ proteinase K (PK), were permeabilized by addition of 6.25 µl of prediluted digitonin (Calbiochem) to final concentrations of 0–2 mg of digitonin per mg of cellular protein. Samples were mixed by vortex and incubated for 10 min at 37°C. To terminate the PK reaction, 100 mM phenylmethylsulfonyl fluoride (PMSF) was added to all samples. Proteins were collected by trichloroacetic acid (TCA) precipitation and analyzed by western blot.

### Alkaline carbonate extraction of membrane proteins

Aliquots containing equal amounts of *L. infantum* promastigotes (5×10^8^) were pelleted and suspended in 2 ml of either 10 mM Tris-HCl pH 7.4, 10 mM Tris-HCl 0.5 M NaCl pH 7.4, or 0.1 M Na_2_CO_3_ pH 11.5 (a cocktail of protease inhibitors was added to all samples). Parasite suspensions were disrupted by sonication, incubated for 30 min at 4°C and sedimented by ultracentrifugation for 60 min at 100,000×*g*. Soluble and insoluble fractions obtained in both procedures were analyzed by western blot.

### Expression and purification of recombinant *Li*ΔTXN3

*LiTXN3* ORF lacking the sequence that encodes the C-terminal hydrophobic tail (*LiΔTXN3*), was PCR-amplified with PWO polymerase (Roche) using the primers 5′-cgcgcacatATGGAGCCCAACTTCTTCAAC-3′ and caccgctcgagTCAACGGGTGTAGTCCAAA-3′ (clamp sequences in lower case; restriction sites underlined). The PCR product was cloned into the *Nde*I-*Xho*I restriction sites of the pET28a expression vector (Novagen), so that *Li*ΔTXN3 could be expressed as a fusion protein carrying an N-terminal tail of six histidines in *Escherichia coli* BL21 strain. Protein expression was induced for 3 h at 37°C in the presence of 50 µg ml^−1^ kanamycin and 0.1 mM isopropyl-*β*-D-thiogalactopyranoside (IPTG) following the protocol detailed before [25]. Bacteria were then pelleted, suspended in 500 mM NaCl, 20 mM Tris-HCl pH 7.6, disrupted by sonication and centrifuged at 10,000×*g* for 30 min at 4°C. The supernatant was applied to a His Bind resin (Novagen) column and the recombinant protein eluted with an imidazole gradient (5 to 1000 mM) at a flow rate of 2.5 ml min^−1^. Fractions confirmed to contain the protein by SDS-PAGE were pooled, applied to PD-10 columns (Amersham) and eluted with PBS.

### Enzymatic and kinetics assays

For the insulin reduction assay, a 12.5 mg ml^−1^ insulin solution was freshly prepared in 100 mM potassium phosphate, 2 mM EDTA, pH 7.0. Reaction mixtures were prepared in 100 mM potassium phosphate, 2 mM EDTA, pH 7.0, containing 125 µM insulin, 5 µM of purified recombinant TXN and different reducing systems: i) 330 µM dithiothreitol (DTT), ii) 300 µM NADPH, 1 U ml^−1^ of *T. cruzi* trypanothione reductase (TR), 100 µM trypanothione disulfide (TS_2_, Bachem), iii) 300 µM NADPH, 2 U ml^−1^ of yeast glutathione reductase (Sigma), 500 µM glutathione (Sigma), iv) 300 µM NADPH, 2 U ml^−1^ of *E. coli* thioredoxin reductase (Sigma), 5 µM *E. coli* thioredoxin (Sigma) and v) 200 µM NADH, 0.5 U ml^−1^ porcine lipoamide dehydrogenase (Sigma), 100 µM dihydrolipoamide (Sigma). The increase in turbidity due to the formation of insoluble insulin B chain was followed at 650 nm. For assaying *Li*ΔTXN3 reduction by trypanothione disulfide, several reaction mixtures were prepared in 100 mM potassium phosphate, 2 mM EDTA, pH 7.0, which contained 300 µM NADPH, 1 U ml^−1^ *T. cruzi* TR, increasing concentrations of TS_2_ (from 25 to 100 µM) and 5 µM *Li*ΔTXN3. The assay mixtures were preincubated until a constant baseline was obtained and the reaction was started by adding 125 µM insulin. The absorption decrease was followed at 340 nm. Routine determination of peroxidase reduction by TXN was performed according to Nogoceke *et al.* [2]. Briefly, reaction mixtures were prepared in a total volume of 300 µl of 50 mM Tris-HCl, 1 mM EDTA, pH 7.6, containing 300 µM NADPH, 1 U ml^−1^ *T. cruzi* TR, 100 µM trypanothione disulfide (TS_2_, Bachem), 5 µM TXN and 5 µM of N-terminal His tagged *L. infantum* peroxidases: i) *Li*mTXNPx (Gene ID LinJ23_V3.0050) [25], ii) *Li*cTXNPx1 (Gene ID LinJ15_V3.1140) [10], iii) *Li*cTXNPx2 (Gene ID LinJ15_V3.1120) [10], and (iv) *Li*nsGPXA1 (Gene ID LinJ26_V3.0770; Teixeira F, Castro H, Tomás AM, unpublished). After a 10 min preincubation period reactions were started by addition of 70 µM hydrogen peroxide (H_2_O_2_, Sigma) and NADPH consumption was followed at 340 nm. All reactions were performed at 25°C and monitored with a Shimadzu UV-2401 PC spectrophotometer (Shimadzu Corporation).

### Phylogenetic analysis of *TXN* sequences

*TXN* sequences were obtained at the TriTrypDB (http://tritrypdb.org/tritrypdb/), except for the *C. fasciculata* sequences, which were recovered from the NCBI webpage (http://www.ncbi.nlm.nih.gov/nuccore). TXNs designation follows the suggested genetic nomenclature for *Trypanosoma* and *Leishmania* [48], mentioning the species abbreviation and the sequence number. Systematic names attributed by TriTrypDB to each gene are listed next (May 2010). *LbTXN1a*, LbrM29_V2.1240; *LbTXN1b*, LbrM29_V2.1230; *LbTXN2*, LbrM29_V2.1220; *LbTXN3*, LbrM31_V2.2190; *LmTXN1*, LmjF29.1160; *LmTXN2*, LmjF29.1150; *LmTXN3*, LmjF31.1960; *LmTXN4*, LmjF31.1970; *LmxTXN1*, LmxM08_29.1160; *LmxTXN2*, LmxM08_29.1150; *LmxTXN3*, LmxM30.1960; *LmxTXN4*, LmxM30.1970; *LiTXN1*, LinJ29_V3.1250; *LiTXN2*, LinJ29_V3.1240; *LiTXN3*, LinJ31_V3.2000; *LiTXN4*, LinJ31_V3.2010; *TbTXN1a*, Tb927.3.3780; *TbTXN1b*, Tb927.3.3760; *TbTXN2*, Tb927.3.5090; *TgTXN1a*, Tbg972.3.4110; *TgTXN1b*, Tbg972.3.4090; *TgTXN2*, Tbg972.3.5720; *TcTXN1*, Tc00.1047053509997.30; *TcTXN2*, Tc00.1047053509997.20; *TcoTXN1*, TcIL3000.0.52740; *TcoTXN2*, TcIL3000.0.52730; *TvTXN1*, TvY486_0303158; *TvTXN2*, TvY486_0303156 (note that some are temporary names). In the case of *C. fasciculata* sequences the accession numbers are as follows: *Cf*TXN1, AF084456; *Cf*TXN2, AF055986). Nucleotide sequences were translated, aligned at the amino acid level with ClustalW (www.ebi.ac.uk/Tools/clustalw/) and the resulting alignments used as a guide to obtain the final nucleotide alignment. Phylogenetic trees were constructed by the MEGA4 program [29].

### Statistical analysis

Statistical significance was analyzed by independent-samples t-test, using SPSS (Version 16.0).

## Supporting Information

Text S1 Sequence analysis of TXN-like proteins predicted in the *L. infantum* genome that do not specify active tryparedoxins.
(0.17 MB PDF)

Text S2 Exchange of Arg40 by a Ser does not allow *Li*ΔTXN3 to recover TXN activity.
(0.13 MB PDF)

Figure S1 Sequence analysis of *Li*TXN4, *Li*TXN5, *Li*TXN6 and *Li*TXN7. A. Nucleotide and deduced amino acid sequence of *LiTXN4*. Residues in the grey background represent the Trp-Cys-Pro-Pro-Cys-Arg active site signature of TXNs. The start and stop codons are underlined, showing that *LiTXN4* is a pseudogene. B-D. Sequence alignment of *Li*TXN5 (B), *Li*TXN6 (C) and *Li*TXN7 (D) with *Li*TXN1 and *Li*TXN2 (the latter two considered here as a distinct subgroup). Strict identity across all sequences is represented with white letters on a black background. Similarity across subgroups is marked with black frames. Residue similarity in each subgroup is indicated with bold letters. Differences between subgroups are marked with black frames and grey background. The TXN active site motif is highlighted with a bar. Residues involved in the hydrogen bond system are marked with stars and those implicated in reaction with trypanothione with arrowheads. The acidic area, important for interaction with 2-Cys PRXs, is marked with diamonds. The C-terminal hydrophobic tail of *Li*TXN5, predicted to specify a transmembrane domain (by bioinformatics analysis with TMpred and TopPred), is marked on light grey background. Sequence numbering refers to *Li*TXN1. The secondary structural elements (β_1–7_ = beta strands; α_1–4_ = alpha helices; η_1–3_ = 310 helices; TT = turns) deduced for *Li*TXN1, according to the crystal structure of recombinant *C. fasciculata* TXN1 (PDB ID 1QK8) is shown in D. The predicted amino acidic sequences of the *Li*TXN7 counterparts in *T. brucei* (*Tb*TXN3) and *T. cruzi* (*Tc*TXN3) (Gene IDs: Tb927.10.3970 and Tc00.1047053506959.20, respectively, according to the TriTrypDB) are also aligned in D.
(0.41 MB PDF)

Figure S2 Analysis of *Li*ΔTXN3R40S enzymatic activity. A. Formation of the insoluble insulin B chain (monitored at 650 nm) by DTT-reduced *Li*ΔTXN3 (filled squares) or *Li*ΔTXN3R40S (open squares), or by NADPH/TR/T(SH)_2_- reduced *Li*ΔTXN3 (filled circles) or *Li*ΔTXN3R40S (open circles). Negative controls were performed in the same reaction mixtures [either with DTT or with the TR/T(SH)_2_ redox system] without any addition of TXN (triangles). B. Reduction of *Li*ΔTXN3R40S by T(SH)_2_ followed by monitoring NADPH consumption at 340 nm. The reaction systems contained NADPH, TR and T(SH)_2_ as reductants for TXN and insulin as final electron acceptor. The TXNs tested were *Li*ΔTXN3 (filled squares) and *Li*ΔTXN3R40S (open squares). Negative control contains no TXN (triangles). C. Reduction of TXN-dependent peroxidases by *Li*ΔTXN3R40S. Reaction mixtures consisted in NADPH, TR, T(SH)_2_, recombinant TXN [either *Li*ΔTXN3 (closed symbols) or *Li*ΔTXN3R40S (open symbols)] and four different peroxidases, namely *Li*mTXNPx (triangles), *Li*cTXNPx1 (squares), *Li*cTXNPx2 (diamonds) and *Li*nsGPXA1 (circles). Reactions were initiated by addition of H_2_O_2_ and NADPH consumption was followed at 340 nm.
(0.10 MB PDF)
