# Supplementary material for: Mitochondrial Redox Metabolism in Trypanosomatids Is Independent of Tryparedoxin Activity
Source: PLoS One. 2010 Sep 8;5(9):e12607. doi: 10.1371/journal.pone.0012607 (PMC2935891; doi:10.1371/journal.pone.0012607)
Supplement: Text S1 — Sequence analysis of TXN-like proteins predicted in the L. infantum genome that do not specify active tryparedoxins. (0.17 MB PDF) [file pone.0012607.s001.pdf]

The *L. infantum* genome contains seven *TXN*/*TXN*-like open-reading frames. Three of these code for the fully active cytosolic and mitochondrial tryparedoxins, *Li*TXN1 and *Li*TXN2, respectively [1], as well as for the *Li*TXN3 enzyme characterized in this manuscript (see main text). The amino acid sequences predicted for the other open reading frames (*LiTXN4* to *LiTXN7*) substitute residues that are critical for TXN function. This should hamper TXN activity in those molecules as experimentally demonstrated for *Li*TXN5 (see below). The major sequence alterations precluding *Li*TXN4, *Li*TXN5, *Li*TXN6 and *Li*TXN7 from behaving as TXNs and, as such substitute for *Li*TXN2 in the *L. infantum* mitochondrion, are discussed next.

LiTXN4

Due to the occurrence of two insertions in the respective nucleotide sequence, *LiTXN4* is a pseudogene in *L. infantum* (Figure S1A). In *L. major,* however, the corresponding sequence codes for a functional protein. In *L. braziliensis* this sequence is missing.

LiTXN5

The *LiTXN5* open reading frame encodes a protein with a theoretical molecular weight of 30.4 kDa. When compared to typical TXNs, *Li*TXN5 shows two distinct features, i) a long middle amino acidic insertion (of 90 residues) that might affect the thioredoxin fold and ii) similarly to *Li*TXN3, a C-terminus hydrophobic stretch specifying a transmembrane domain (Figure S1B). *LiTXN5* shows no obvious counterparts in *Trypanosoma* spp. Inactivity of *Li*TXN5 is predicted because:

i) The hydrogen bond network, required to support the reactivity of the active site sulfurs [2], is incomplete owing to replacement of Tyr80 by a non-identical Phe residue (*Li*TXN1 numbering).

ii) *Li*TXN5 does not conserve Arg44 nor the negative residue at position 72, which along with Pro110 and Arg128 (substituted in *Li*TXN5 by an equivalent Lys), are required for binding to trypanothione. Arginine 44 is conserved in all enzymes with known TXN activity and is important for TXN catalysis. Illustrating this, substitution of Arg44 by an acidic residue in the *Cf*TXN2 enzyme resulted in loss of activity [3].

iii) The absence of the negative charge at site 72 should also compromise contact of *Li*TXN5 with 2-Cys PRXs, as suggested by Budde *et al.* [4]. The interaction with these peroxidases should, in addition, be complicated by the Ser to His substitution at position 38.

iv) Finally, reactivity of *Li*TXN5 with nsGPx is likely to be conditioned by the absence of Glu107 [5].

The prediction that *Li*TXN5 is inactive as a TXN was experimentally confirmed using 6HisΔ*Li*TXN5 (a truncated version of the protein lacking the C-terminal hydrophobic tail), produced similarly to 6HisΔ*Li*TXN3 (see main text). For this, 6HisΔ*Li*TXN5 was PCR amplified using the primers 5'-ccgcgcacatATGTCGGGTTTGACGAAG-3' and 5'-caccgctcgagCTATTCTCGCCAGCCCTCGGT-3' (clamp sequences in lower case; restriction sites underlined) and cloned into the *Nde*I-*Xho*I restriction sites of the pET28a expression vector (Novagen). When tested in the Holmgren assay [6], using either DTT, the TR/T$(SH)_2$ system, dihydrolipoamide, thioredoxin or glutathione as electron donors, 6HisΔ*Li*TXN5 was always found inactive. When insulin was replaced by different 2-Cys PRXs or by nsGPX (and peroxides) the enzyme was also inactive. Thus, 6HisΔ*Li*TXN5 lacks TXN and oxidoreductase activity.

LiTXN6

LiTXN6 encodes a protein with a predicted molecular weight of 41.2 kDa. This length (more than the double of typical TXNs) is due to several insertions in the amino acidic sequence. These insertions, along with the non-preservation of important sequence features, should preclude *Li*TXN6 from functioning as a TXN (Figure S1C). The most striking example of these changes is the replacement of the proximal redox active Cys (Cys40, *Li*TXN1 numbering) by a Ser residue. When induced in recombinant *Cf*TXN2, this amino substitution completely abolished activity [3], thus on its own it would exclude *Li*TXN6 from behaving as an active TXN. *LiTXN6* has no equivalent among *Trypanosoma* spp.

LiTXN7

The *LiTXN7* nucleotide sequence encodes a protein with a theoretical molecular weight of 65.5 kDa. *LiTXN7* has orthologue sequences in *Trypanosoma*, which are designated here as *TXN3*. Translation of this ORF indicates that, if expressed, *Li*TXN7 is a hybrid protein made up of an N-terminal region constituting ¾ of the protein and carrying no domains of known function, and a C-terminal portion containing a TXN-like sequence followed by a stretch of 71 amino acids without any recognizable signature (Figure S1D). This second region presents a higher degree of similarity to *TXN3* of the other

trypanosomatids than the N-terminal which is poorly conserved between *Leishmania* and *Trypanosoma* spp. *Li*TXN7 does not specify any membrane anchor region. Lack of TXN activity in *Li*TXN7 can be inferred from analysis of it amino acidic sequence. Importantly, many of the observed changes are shared with *Li*TXN5 which was shown experimentally to be inactive.

i) The hydrogen bond system supporting reactivity of the redox-active cysteines is incomplete in *Li*TXN7. This is due to absence of both Ser36 and Thr47 (*Li*TXN1 numbering). In fact, substitution of Ser36 by the non-identical Gly residue should, *per se*, affect TXN activity. Ser36 was reported to be required both to fix Cys43 in its correct position in the three dimensional structure of TXNs, and to promote activation of the active site cysteines [2].

ii) Interaction of *Li*TXN7 with trypanothione ought to be hindered by the non-conservation of both Arg44 and the negatively charged residue at position 72. Of notice, although *Li*TXN7 maintains Arg128, a residue that is also required for this interaction, the *Trypanosoma* counterparts do not, supporting the idea that these molecules do not function as TXNs in trypanosomatids.

iii) The absence of a negatively charged residue at position 72 should also hamper contact of *Li*TXN7 with the peroxidases.

iv) The TXN-domain of *Li*TXN7 lacks 24 residues that form the β5, η3 and α3 secondary structures and this may affect the thioredoxin fold.

References

1. Castro H, Sousa C, Novais M, Santos M, Budde H, et al. (2004) Two linked genes of *Leishmania infantum* encode tryparedoxins localised to cytosol and mitochondrion. Mol Biochem Parasitol 136: 137-147.
2. Hofmann B, Budde H, Bruns K, Guerrero SA, Kalisz HM, et al. (2001) Structures of tryparedoxins revealing interaction with trypanothione. Biol Chem 382: 459-471.
3. Steinert P, Plank-Schumacher K, Montemartini M, Hecht HJ, Flohé L (2000) Permutation of the active site motif of tryparedoxin 2. Biol Chem 381: 211-219.
4. Budde H, Flohé L, Hecht HJ, Hofmann B, Stehr M, et al. (2003) Kinetics and redox-sensitive oligomerisation reveal negative subunit cooperativity in tryparedoxin peroxidase of *Trypanosoma brucei brucei*. Biol Chem 384: 619-633.
5. Melchers J, Diechtierow M, Feher K, Sinning I, Tews I, et al. (2008) Structural basis for a distinct catalytic mechanism in *Trypanosoma brucei* tryparedoxin peroxidase. J Biol Chem 283: 30401-30411.
6. Holmgren A (1979) Thioredoxin catalyzes the reduction of insulin disulfides by dithiothreitol and dihydrolipoamide. J Biol Chem 254: 9627-9632.
