# Supplementary material for: Mitochondrial Redox Metabolism in Trypanosomatids Is Independent of Tryparedoxin Activity
Source: PLoS One. 2010 Sep 8;5(9):e12607. doi: 10.1371/journal.pone.0012607 (PMC2935891; doi:10.1371/journal.pone.0012607)
Supplement: Text S2 — Exchange of Arg40 by a Ser does not allow LiΔTXN3 to recover TXN activity. (0.13 MB PDF) [file pone.0012607.s002.pdf]

The *Trypanosoma* TXN2 enzymes (e.g. *Tb*TXN2 and *Tc*TXN2) share with *Li*TXN3 all sequence alterations that potentially impair TXN activity (Figure 4 of the main manuscript). The exception is a Ser residue at position 40 (*Li*TXN3 numbering), present in the trypanosome enzymes, but replaced by an Arg in *Li*TXN3. The Ser at this position is present in all enzymes with known TXN activity and is in the vicinity of an acidic area proposed to be important for TXN interaction with 2-Cys PRXs [1]. To investigate whether the presence of a Ser residue at position 40 would allow *Li*TXN3 to recover its activity as a TXN, we produced and purified from *E. coli* the recombinant *Li*ΔTXN3R40S mutein by site directed mutagenesis. Enzymatic assays performed with this enzyme showed that it behaves like the wild type *Li*ΔTXN3 in what concerns its ability i) to reduce insulin (Figure S2A), ii) to be reduced by trypanothione (Figure S2B), and iii) to electron fuel 2-Cys PRXs and nsGPXs (Figure S2C). In other words, the impaired activity of *Li*TXN3 is not determined by the lack of a Ser residue at position 40. Rather it results from the accumulation of several other substitutions, which are also present in the TXN2 sequences of *Trypanosoma*. Accordingly, it is reasonable to assume that the trypanosome enzymes also display deficient TXN activity.

References

1. Budde H, Flohé L, Hecht HJ, Hofmann B, Stehr M, et al. (2003) Kinetics and redox-sensitive oligomerisation reveal negative subunit cooperativity in tryparedoxin peroxidase of *Trypanosoma brucei brucei*. Biol Chem 384: 619-633.
