# Supplementary material for: Mitochondrial Redox Metabolism in Trypanosomatids Is Independent of Tryparedoxin Activity
Source: PLoS One. 2010 Sep 8;5(9):e12607. doi: 10.1371/journal.pone.0012607 (PMC2935891; doi:10.1371/journal.pone.0012607)
Supplement: Figure S1 — Sequence analysis of LiTXN4, LiTXN5, LiTXN6 and LiTXN7. A. Nucleotide and deduced amino acid sequence of LiTXN4. Residues in the grey background represent the Trp-Cys-Pro-Pro-Cys-Arg active site signature of TXNs. The start and stop codons are underlined, showing that LiTXN4 is a pseudogene. B-D. Sequence alignment of LiTXN5 (B), LiTXN6 (C) and LiTXN7 (D) with LiTXN1 and LiTXN2 (the latter two considered here as a distinct subgroup). Strict identity across all sequences is represented with white letters on a black background. Similarity across subgroups is marked with black frames. Residue similarity in each subgroup is indicated with bold letters. Differences between subgroups are marked with black frames and grey background. The TXN active site motif is highlighted with a bar. Residues involved in the hydrogen bond system are marked with stars and those implicated in reaction with trypanothione with arrowheads. The acidic area, important for interaction with 2-Cys PRXs, is marked with diamonds. The C-terminal hydrophobic tail of LiTXN5, predicted to specify a transmembrane domain (by bioinformatics analysis with TMpred and TopPred), is marked on light grey background. Sequence numbering refers to LiTXN1. The secondary structural elements (β1–7 = beta strands; α1–4 = alpha helices; η1–3 = 310 helices; TT = turns) deduced for LiTXN1, according to the crystal structure of recombinant C. fasciculata TXN1 (PDB ID 1QK8) is shown in D. The predicted amino acidic sequences of the LiTXN7 counterparts in T. brucei (TbTXN3) and T. cruzi (TcTXN3) (Gene IDs: Tb927.10.3970 and Tc00.1047053506959.20, respectively, according to the TriTrypDB) are also aligned in D. (0.41 MB PDF) [file pone.0012607.s003.pdf]

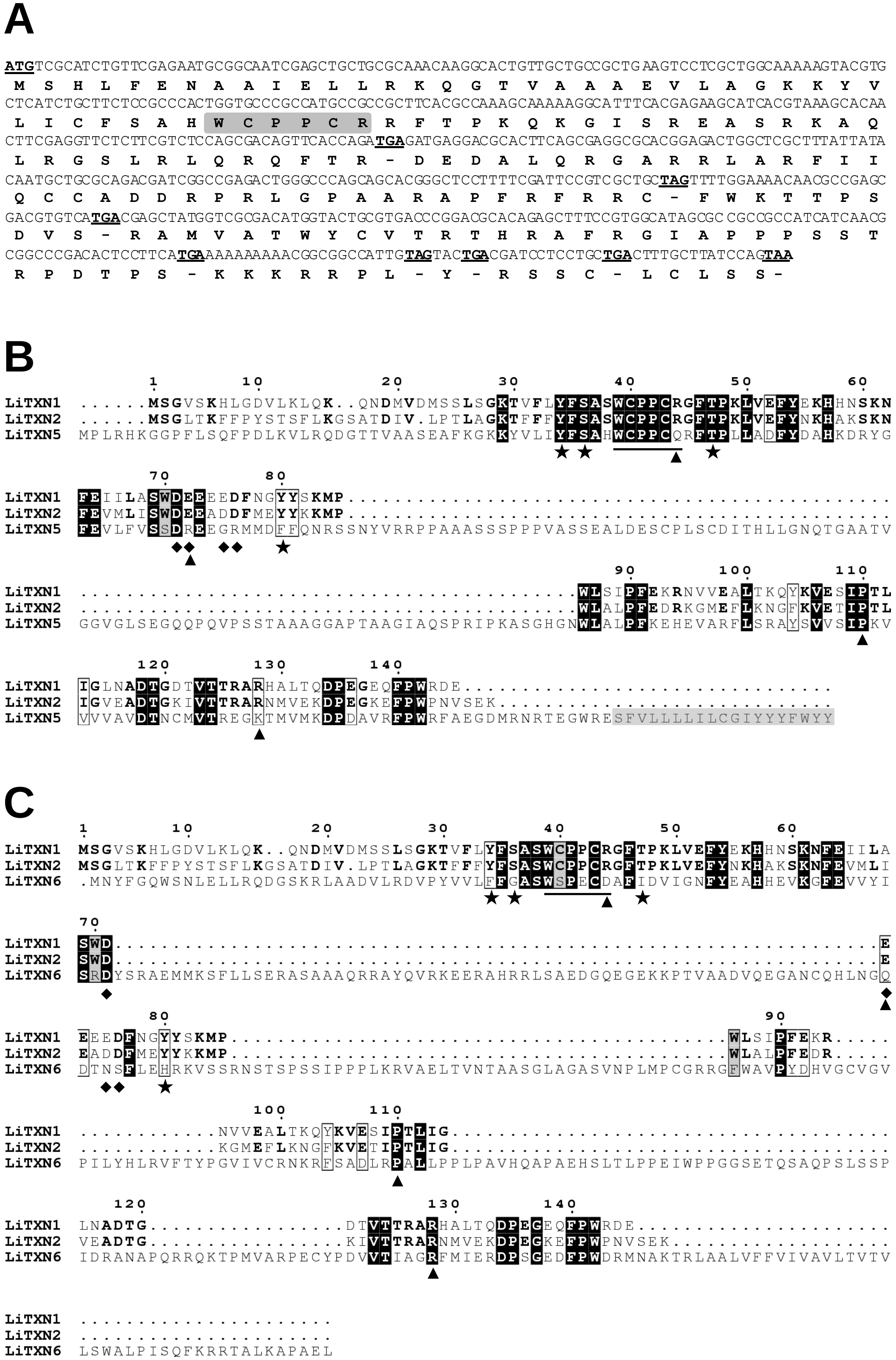
A
ATGTCGCATCTGTTCGAGAATGCGGCAATCGAGCTGCTGCGCAAACAAGGCACTGTTGCTGCCGCTGAAGTCCTCGCTGGCAAAAAGTACGTG
M S H L F E N A A I E L L R K Q G T V A A A E V L A G K K Y V
CTCATCTGCTTCTCCGCCCACTGGTGCCCGCCATGCCGCCGCTTCACGCCAAAGCAAAAAGGCATTTCACGAGAAGCATCACGTAAAGCACAA
L I C F S A H W C P P C R R F T P K Q K G I S R E A S R K A Q
CTTCGAGGTTCTCTTCGTCTCCAGCGACAGTTCACCAGATGAGATGAGGACGCACTTCAGCGAGGCGCACGGAGACTGGCTCGCTTTATTATA
L R G S L R L Q R Q F T R - D E D A L Q R G A R R L A R F I I
CAATGCTGCGCAGACGATCGGCCGAGACTGGGCCCAGCAGCACGGGCTCCTTTTCGATTCCGTCGCTGCTAGTTTTGGAAAACAACGCCGAGC
Q C C A D D R P R L G P A A R A P F R F R R C - F W K T T P S
GACGTGTCATGACGAGCTATGGTCGCGACATGGTACTGCGTGACCCGGACGCACAGAGCTTTCCGTGGCATAGCGCCGCCGCCATCATCAACG
D V S - R A M V A T W Y C V T R T H R A F R G I A P P P S S T
CGGCCCGACACTCCTTCATGAAAAAAAAAACGGCGGCCATTGTAGTACTGACGATCCTCCTGCTGACTTTGCTTATCCAGTAA
R P D T P S - K K K R R P L - Y - R S S C - L C L S S -
B
LiTXN1 ......MSGVSKHLGDVLKLQK..QNDMVDMSSLSGKTVFLYFSASWCPPCRGFTPKLVEFYEKHHNSKN
LiTXN2 ......MSGLTKFFPYSTSFLKGSATDIV.LPTLAGKTFFFYFSASWCPPCRGFTPKLVEFYNKHAKSKN
LiTXN5 MPLRHKGGPFLSQFPDLKVLRQDGTTVAASEAFKGKKYVLIYFSAHWCPPCQRFTPLLADFYDAHKDRYG
LiTXN1 FEIILASWDEEEEDFNGYYSKMP..
LiTXN2 FEVMLISWDEEADDFMEYYKKMP..
LiTXN5 FEVLFVSSDREEGRMMDFFQNRSSNYVRRPPAAASSSPPPVASSEALDESCPLSCDITHLLGNQTGAATV
LiTXN1 ..................................WLSIPFEKRNVVEALTKQYKVESIPTL
LiTXN2 ..................................WLALPFEDRKGMEFLKNGFKVETIPTL
LiTXN5 GGVGLSEGQQPQVPSSTAAAGGAPTAAGIAQSPRIPKASGHGNWLALPFKEHEVARFLSRAYSVVSIPKV
LiTXN1 IGLNADTGDTVTTRARHALTQDPEGEQFPWRDE..................
LiTXN2 IGVEADTGKIVTTRARNMVEKDPEGKEFPWPNVSEK...............
LiTXN5 VVVAVDTNCMVTREGKTMVMKDPDAVRFPWRFAEGDMRNRTEGWRESFVLLLLILCGIYYYFWYY
C
LiTXN1 MSGVSKHLGDVLKLQK..QNDMVDMSSLSGKTVFLYFSASWCPPCRGFTPKLVEFYEKHHNSKNFEIILA
LiTXN2 MSGLTKFFPYSTSFLKGSATDIV.LPTLAGKTFFFYFSASWCPPCRGFTPKLVEFYNKHAKSKNFEVMLI
LiTXN6 .MNYFGQWSNLELLRQDGSKRLAADVLRDVPYYVLFFGASWSPECDAFIDVIGNFYEAHHEVKGFEVVYI
LiTXN1 SWD...E
LiTXN2 SWD...E
LiTXN6 SRDYSRAEMMKSFLLSERASAAAQRRAYQVRKEERAHRRLSAEDGQEGEKKPTVAADVQEGANCQHLNGQ
LiTXN1 EEEDFNGYYSKMP.............................WLSIPFEKR.....
LiTXN2 EADDFMEYYKKMP.............................WLALPFEDR.....
LiTXN6 DTNSFLEHRKVSSRNSTSPSSIPPPLKRVAELTVNTAASGLAGASVNPLMPCGRRGFWAVPYDHVGCVGV
LiTXN1 ...........NVVEALTKQYKVESIPTLIG..................................
LiTXN2 ...........KGMEFLKNGFKVETIPTLIG..................................
LiTXN6 PILYHLRVFTYPGVIVCRNKRFSADLRPALLPPLPAVHQAPAEHSLTLPPEIWPPGGSETQSAQPSLSSP
LiTXN1 LNADTG................DTVTTRARHALTQDPEGEQFPWRDE..................
LiTXN2 VEADTG................KIVTTRARNMVEKDPEGKEFPWPNVSEK...............
LiTXN6 IDRANAPQRRQKTPMVARPECYPDVVTIAGRFMIERDPSGEDFPWDRMNAKTRLAALVFFVIVAVLTVTV
LiTXN1 ....
LiTXN2 ....
LiTXN6 LSWALPISQFKRRTALKAPAEL

D

```
LiTXN1  ......................................................................
LiTXN2  ......................................................................
LiTXN7  MLAFTITSTEQERTVKRLKVDSPSASLECEDDGENSCSS..........VPHIAEANPALESAQRTPALS
TbTXN3  MVGASSKRPREHETAVEP.......ILAGSFSGGNVSKK.........SFNGTVVAETDNKSLGVTATL.
TcTXN3  MHSTEKRGREETEVAAVPTVHETEGVVVVGKGDDDVRKKKKRPHLENDADNNNNNSTTNNNNNDVTDTLD

LiTXN1  ......................................................................
LiTXN2  ......................................................................
LiTXN7  ........KHADTPATEAVNGVSPEVSPANSSRSRHSSDDDDDGKYEKDLSDDHLSSPVARQFNGDTWRV
TbTXN3  .......LRDVDFNSQSSMKRSVSNNSNMVGSQNDDSNDECEGEVCELFKSPAQLGGKLQVGVDSRPD..
TcTXN3  AHKKFAGLREERPQQNEPLQHPPSVNEVSVDGRGETDGSEVGANDVDVEEPTLFFGPTHAARDEKRSDGS

LiTXN1  ......................................................................
LiTXN2  ......................................................................
LiTXN7  TRTPTGSAVKATTAITGAAAVPPPPITTNGAITTLRSLGQLQTLVSSTNTTSAKD...........KVHP
TbTXN3  .....VPVSNGQKTLLHAECTNSTAS.VDGAITTLHTLQQLKELFHTPPVQLEAQ...........AKAP
TcTXN3  GVDLRCVASGGQNQRPDAAHDGCRAPHTVGAVTTLYSLRQLEELIGSAPLPIAHAGVLNVTPSSSSSAPP

LiTXN1  ......................................................................
LiTXN2  ......................................................................
LiTXN7  SLRLKPGETVMLLLHFDETSLRSLINGATTELSQMRVFTLNLAKLPPHDYEAAPEKRLSAGGCTSSPDAS
TbTXN3  LLQLCAGETVFVVLHSNKEEIEGMLGNVTKKLSACRIFCLDLSALPLAERSGA........GAQITPNSA
TcTXN3  SLRLRAGETVLIMLHRCKGEAGVLPQHTTEQLSNCRIFLLDLGELPPCEYEEK........SDVACPNGH

LiTXN1  ......................................................................
LiTXN2  ......................................................................
LiTXN7  DRARRGGSTATPDADVTQVTVQDTEDEDEEELVPAVHPVAGSTAAAAGTSTIAEKECLPRASVSTGGALA
TbTXN3  SVDRVG.........DVILQRVTK.ILDLNSLLNGTPSAE.......................GPSQSLS
TcTXN3  DERGVATGFLQLPPTDTLLSLASQGAHDMEHVMRRASSLL.......................CVDEVLN

LiTXN1  ......................................................................
LiTXN2  ......................................................................
LiTXN7  LDEAATVSSLSGDLHHASDVISDKLHQMLAVDTILNPHASQQQRGHGSGDGGAAATGKKEPLLFPAMIVW
TbTXN3  IKHSSAGAQV............................EFEN..VASSAQSSAKDEICHHELTLPSMVMW
TcTXN3  ANDADSSKSQ............................TSEKGGGDENASADATHSCKREELALPALVMW

                                                                   1        10
LiTXN1  ...........................................................MSGVSKHLGDV
LiTXN2  ...........................................................MSGLTKFFPYS
LiTXN7  RAAGAPREEYPPAPPQTYSSQPPSTPAELFSDRLS..AQGGPLVVKEATGVDQVHSLTLFRPVFTMEHLF
TbTXN3  RVAGGPVDEYGDITAMGSYEAIQSEVGSVPP.......YGKPLVVKELQTVDQLHSLSLLAPVYTVEHLL
TcTXN3  RAEGGLADEYCYPKEKGKNDSHANNHDNPQPPLAGRYHHGRPLVVKQLTSLDQLHSFSLLTPVYTVTHFL

LiTXN1   β1     TT β2  η1   β3           α1                 β4        η2  α2
              20        30        40        50        60        70
LiTXN1  LKLQK..QNDMVDMSSLSGKTVFLYFSASWCPPCRGFTPKLVEFYEKHHNSKNFEIILASWDEEEEDFNG
LiTXN2  TSFLKGSATDIV.LPTLAGKTFFFYFSASWCPPCRGFTPKLVEFYNKHAKSKNFEVMLISWDEEADDFME
LiTXN7  RTLVRTVAPSFAARGTGEKSRTVLYLGASWCPPCMRFVREMPQLMREDLPSSVVCTLKADMDLAKPIYD.
TbTXN3  RKIGETVLFPPGNDT.PSGTHTFIYMGASWCPPCMRVVASLPTMMKEGFPHSFACVVKADMDFAEPLYK.
TcTXN3  RTVCEKALFPDDPAAGASGTRSLIYFGASWCPPCMRIVHALPTMMKEDFPSNMTCSVKADMDLATPLFE.

LiTXN1         β5   η3     α3           β6 TT......................T β7
        80        90        100       110                      120
LiTXN1  YYSKMPWLSIPFEKRNV.VEALTKQYKVESIPTLIGLNAD......................TGDTVTTR
LiTXN2  YYKKMPWLALPFEDRKG.MEFLKNGFKVETIPTLIGVEAD......................TGKIVTTR
LiTXN7  YF........................KVEIIPTFVVLDNEVLMKCYETVRRQQSSAAGCSGGGGASQSDY
TbTXN3  FF........................GVEIIPTFILLNNDVLKGAGDWSAFLK........GSGKCMGTC
TcTXN3  FF........................GVEIIPTFVILDNESLRTAGDWKALR..........SCSISEEA

LiTXN1    α4
          130       140
LiTXN1  ARHALTQDPEGEQFPWRDE......................
LiTXN2  ARNMVEKDPEGKEFPWPNVSEK...................
LiTXN7  ARAIQEAFSKAELGRLQNSNRQLVSTFISKHSQALSFDEDF
TbTXN3  LEQLREGLRRSKMGQIQNSKVPLIQSFIDNHTKGLSFDEEF
TcTXN3  LSQIYEGLRRSELGRIQNSQRVTVRTFIERHSGMLKFDEDF
```
