# Supplementary figures and images for: Mitochondrial Redox Metabolism in Trypanosomatids Is Independent of Tryparedoxin Activity
Source: PLoS One. 2010 Sep 8;5(9):e12607. doi: 10.1371/journal.pone.0012607 (PMC2935891; doi:10.1371/journal.pone.0012607)

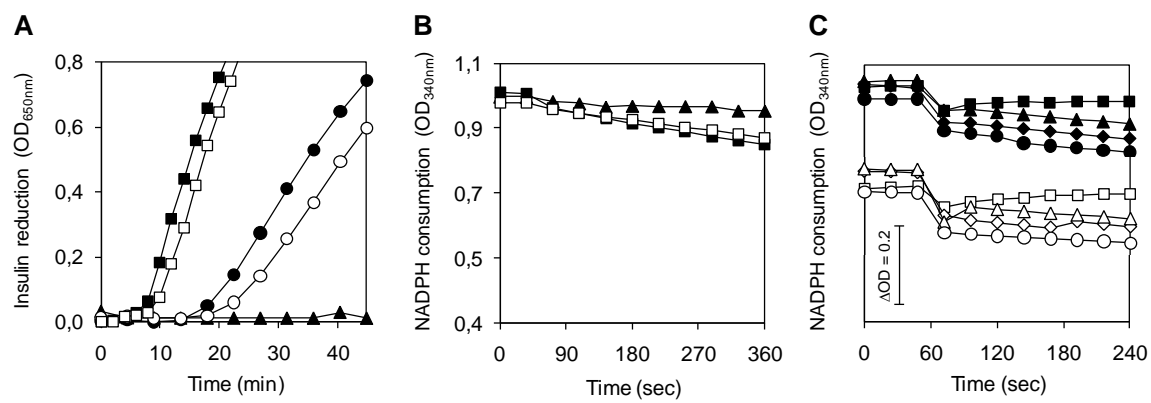

Supplement: Figure S2 — Analysis of LiΔTXN3R40S enzymatic activity. A. Formation of the insoluble insulin B chain (monitored at 650 nm) by DTT-reduced LiΔTXN3 (filled squares) or LiΔTXN3R40S (open squares), or by NADPH/TR/T(SH)2- reduced LiΔTXN3 (filled circles) or LiΔTXN3R40S (open circles). Negative controls were performed in the same reaction mixtures [either with DTT or with the TR/T(SH)2 redox system] without any addition of TXN (triangles). B. Reduction of LiΔTXN3R40S by T(SH)2 followed by monitoring NADPH consumption at 340 nm. The reaction systems contained NADPH, TR and T(SH)2 as reductants for TXN and insulin as final electron acceptor. The TXNs tested were LiΔTXN3 (filled squares) and LiΔTXN3R40S (open squares). Negative control contains no TXN (triangles). C. Reduction of TXN-dependent peroxidases by LiΔTXN3R40S. Reaction mixtures consisted in NADPH, TR, T(SH)2, recombinant TXN [either LiΔTXN3 (closed symbols) or LiΔTXN3R40S (open symbols)] and four different peroxidases, namely LimTXNPx (triangles), LicTXNPx1 (squares), LicTXNPx2 (diamonds) and LinsGPXA1 (circles). Reactions were initiated by addition of H2O2 and NADPH consumption was followed at 340 nm. (0.10 MB PDF) [file pone.0012607.s004.pdf]
